# Peroxisomes in host defense

**DOI:** 10.1371/journal.ppat.1008636

**Published:** 2020-07-02

**Authors:** Francesca Di Cara

**Affiliations:** Department of Microbiology and Immunology-IWK Health Centre- Dalhousie University, Halifax (NS), Canada; Tufts Univ School of Medicine, UNITED STATES

## Introduction

Survival from infection and altered self requires an effective and tightly controlled immune response. Disorders of immunodeficiency or autoimmunity can be directly attributed to imbalances in the immune response [1–3]. It is now evident that changes in the metabolic status of cells and tissues have important and long-overlooked impacts on immunity [4, 5] and immune-related abnormalities [6, 7]. The reprogramming of immune cell metabolism is a regulatory event that governs the nature of the immune response in both health and disease. Studies of the metabolic signals that regulate host defense responses provide new insight into the determinants of immunity and the extent by which metabolic diseases, such as obesity and diabetes, are caused by immune dysfunctions.

Immunometabolic studies so far, focus primarily on glycolysis and oxidative phosphorylation, and have strongly affirmed the importance of these central energy supply pathways to support innate and adaptive immune cell activity and survival [4] [8, 9]. These findings opened avenues of investigation into additional metabolic networks that operate in the immune system, which have yet to be fully explored [4].

Peroxisomes are essential metabolic organelles present in virtually every eukaryotic cell and are important sites of distinct metabolic reactions essential for survival. Recent evidence demonstrated that peroxisomes are required for immune cell development and function [10], regulating key immune response pathways, such as the activation of nuclear factor kappa-light-chain-enhancer of activated B cells (NF-𝜅B) during bacterial infection [11] and mitochondrial antiviral signaling adaptor (MAVS)-mediated antiviral responses [12].

Here, we will summarize the last 10 years of discoveries that led to the recognition that peroxisomes are metabolic mediators of immunity. We will focus on findings that revealed a peroxisome requirement for immunity as well as evidence that defined the functional roles peroxisomes play in immune cell development and activity.

## The peroxisome

Peroxisomes are ubiquitous organelles that regulate the synthesis and turnover of complex lipids. Peroxisomes are the only cellular compartment in eukaryotic cells where the β-oxidation of very-long chain fatty acids (VLCFAs), the synthesis of ether lipids, and the α-oxidation of branch-chained fatty acids takes place. Moreover, peroxisomes contribute to the detoxification of reactive anionic species and the metabolism of polyamines, amino acids, and carbohydrates [13, 14]. Delimited by a single membrane, peroxisomes arise either de novo from the endoplasmic reticulum (ER) [15] or by growth and fission of existing organelles [16]. Conserved *Peroxin* (*Pex*) genes encode proteins required for the formation and maintenance of the cellular peroxisome population [16–18].

Peroxisome metabolic functions are closely related with mitochondrial metabolism. Specifically, both organelles tightly cooperate to control fatty acids β-oxidation [19] and to maintain the oxidative homeostasis [20] in the cell. Until a few years ago, most studies pointed solely to the mitochondrion as a central hub of the immune and inflammatory response [21] against a variety of pathogens. Mitochondria act as a transmission site for different immune signaling events, such as the initiation of the MAVS-proteins–mediated interferon response [22] and the activation of the inflammasome [23]. Also, mitochondria contribute to the metabolic changes that affect immune cell behaviour [24, 25]. For instance, during the early or active phase of an immune response, the cellular metabolic shift from a catabolic to an anabolic state, such as the switch from fatty acids β-oxidation to fatty acid synthesis, is essential to drive the transformation of immune cells from metabolically quiescent (inactive) to a highly active metabolic state (activated) [24]. Although peroxisomes share many features with mitochondria, such as fatty acids β-oxidation, which is important for immune cell regulation (e.g., memory T-cells activation) [26], their unique role as pivotal regulators of cellular and systemic immune responses emerged only recently.

## Peroxisomes regulate host–pathogen interactions

Peroxisomes were first described by a Swedish doctoral student, Johannes Rhodin, in 1954[27]. They were classified as organelles by the cytologist Christian de Duve in 1966 [28]. In 1978, de Duve and Lazarow realized that dysfunction in peroxisome activities affected VLCFA metabolism [29]. In 1989, Lazarow and Moser linked peroxisomal lipid metabolic defects to the insurgence of the cerebro-hepato-renal Zellweger syndrome that is now classified as severe form of peroxisome biogenesis disorder (PBD) [30], a genetic and metabolic condition caused by the deficiency or functional impairment of peroxisomes [31]. Since then, multiple studies of patients with PBD have illuminated the critical role of peroxisomes in human health and development, which revealed further connections between peroxisome dysfunction and other pathologies, such as Alzheimer’s and Parkinson’s diseases, aging, cancer, type 2 diabetes, and heart failure [32–36]. These findings suggested that peroxisomes contribute in different ways to the function, development and survival of different tissues. For instance, peroxisomes are increasingly recognized as producers of distal neurotrophic factors for the survival and function of central and peripheral neurons, responsible for the production of primary bile acids in liver hepatocytes and mediators of muscle function in myocytes [37]. Only in the past 10 years, peroxisomes have been described as regulators of the immune cell functions in response to viral and bacterial elicitors [10–12].

The first report that linked peroxisomes to the immune system was in 1974, by Gilchrist and colleagues, in a study which reported defects in differentiation and function of T cells in clinical cases of cerebro-hepato-renal Zellweger syndrome [38]. A few years later, in 1979, a report by Euguchi and colleagues described that peroxisomes of rat peritoneal macrophages were located proximal to phagosomes during phagocytosis, suggesting a role for the organelle in the phagocytic clearance of pathogens [39]. Despite these early observations, a role for peroxisomes in immunity was long overlooked.

In the past decade, multiple studies demonstrated that peroxisomes, like mitochondria, control immune pathways by producing bioactive metabolites important to drive immune signaling and also by recruiting signaling proteins to their membrane to promote immune pathways activation in response to a stimulus.

The first report was by Dixit and colleagues in 2010 where the authors showed that peroxisomes are essential to trigger interferon-mediated antiviral signaling. This study demonstrating that peroxisomes constitute an antiviral signaling platform and thus contribute to innate immunity was a real breakthrough in the peroxisomal field [12].

The main antiviral signaling pathways depend on the detection of viral proteins, lipids and nucleic acids by pattern recognition receptors (PRRs). Viral proteins are recognized by PRRs such as toll-like receptors (TLR)2/1, TLR2/6, and TLR4, while viral DNA, single-stranded RNA, and double-stranded RNA are recognized by TLR9, TLR7/8, and TLR3. Respectively [40, 41]. Cytosolic receptors such as RIG-I-like receptors (RLRs) are also involved in detecting viral nucleic acids [42]. Binding to viral RNA induces conformational changes in RLRs, which trigger their interaction with MAVS proteins. MAVS is a tail-anchored protein first described on the outer membrane of mitochondria [22]. Downstream signaling pathways activate transcription factors such as NF-𝜅B and interferon regulatory factors (IRFs), leading to production of proinflammatory cytokines and type I or III interferons. Dixit and colleagues reported that the MAVS localize in multiple cellular sites including mitochondria, a specific region of the ER membrane called mitochondrial-associated ER membrane, and peroxisomes [12, 43, 44] to mount an antiviral response. The localization of MAVS to peroxisomes and mitochondria drives different antiviral signaling programs, and peroxisome-associated MAVS seem to activate type III interferon response [12]. Moreover, the same group established that RLR-mediated type III interferon expression can be induced by various viruses, including reoviruses, Sendai viruses, and dengue viruses [45]. More recently, proteomic analysis of peroxisome-enriched fractions from Sendai virus–infected HepG2 cells identified 25 proteins that were previously linked to immune response signaling in virally infected cells compared to noninfected control cells [46], suggesting that peroxisomes are essential signaling platforms that regulate diverse antiviral responses.

## Peroxisomes turn on/off pivotal cellular immune signaling

Multiple studies have further recognized the importance of peroxisomes in cellular innate immune signaling and inflammation. In a recent work, we defined mechanisms by which peroxisomes might control immune cell activities, such as phagocytosis and modulation of immune pathways in response to pathogens [11]. Using the genetic model system *Drosophila melanogaster*, we observed that peroxisomes control phagosome formation and maturation in macrophages and that their intervention is required for elimination of bacterial and fungal pathogens and host survival from the infection. We also demonstrated that the requirement for peroxisome metabolites in phagocytosis is conserved in the murine system. Phagocytosis is a complex process that involves a massive reorganization of the plasma membrane structure and composition. It is well known that phagocytosis is affected by lipid membrane composition and is dependent on the extracellular and intracellular lipid environment. Peroxisome lipid metabolism has an extensive impact on lipid membrane composition, and peroxisome dysfunction leads to an unbalanced pool of cellular lipids necessary to support changes in immune cell membranes, which in turn affect the phagocytic capacity of a cell [10, 47–49]. Membrane properties are indeed modified by changes in their fatty acid and cholesterol content [50]. Peroxisomes regulate intracellular and membrane fatty acid and cholesterol levels [51]. Of notice, the metabolism of polyunsaturated fatty acids (PUFAs) is partly dependent on peroxisomal β-oxidation, and PUFAs are direct modulators of phagocytosis in different phagocytic cells [52–56]. Using genetic and biochemical approaches, we demonstrated that peroxisomes regulate phagocytosis by providing fatty acids such as the PUFA docosahexaenoic acid (DHA) and reactive oxygen species (ROS) to promote the phagosome formation.

Changes in membrane lipid composition during an infection links the process of phagocytosis to the activation of other immune response strategies, such as the activation of inflammatory pathways. The incorporation of DHA into the cell membrane not only affects phagosome formation but, at the same time, alters the composition of lipid nanodomains that control the assembly or expulsion of a variety of transmembrane receptors. This effect impacts the ability of PRRs such as toll-like receptors, TLR2 and TLR4, to activate or inhibit, respectively, canonical proinflammatory signaling [57]. Therefore, the role of peroxisome-derived lipids can directly impact innate immune signaling that regulates differentiation, activation, and inhibition of multiple immune cells. Evidence from studies on the *Drosophila melanogaster* model system also unravelled the involvement of peroxisome metabolism in the regulation of innate immune signaling. Defects in immune signaling through the mitogen-activated protein kinases (MAPKs) cascade and NF-𝜅B were associated with peroxisome dysfunction. Additionally, subsequent evidence in *Drosophila* with dysfunctional peroxisomes in the intestinal epithelium showed heightened susceptibility to enteric bacterial infection and a pronounced intestinal dysbiosis due to an accumulation of cellular free fatty acids [58].

In a study carried out in a mouse model, Vijayan and colleagues probed the immunomodulatory properties of peroxisomes in macrophages [59]. In RAW 264.7 murine macrophage cell lines and in primary alveolar and peritoneal murine macrophages, the induction of peroxisome proliferation by treatments with 4-phenyl butyric acid, a noncanonical peroxisome proliferator, can reduce the expression of lipopolysaccharide (LPS)-induced proinflammatory proteins such as cyclooxygenase (COX-2), tumor necrosis factor alpha (TNF-α), and interleukins 6 (IL-6) and 12 (IL-12). Conversely, a macrophage cell line lacking functional peroxisomes, due to a mutation in *Peroxin14* (Pex14), a gene that encodes for a peroxisomal membrane anchor protein required for peroxisome biogenesis, did not show this reduction in COX2 or any other inflammatory cytokines. The antiinflammatory effect was found to be dependent on peroxisomal β-oxidation activity because the deletions of key peroxisomal β-oxidation enzymes cause hyperexpression of COX2 and TNF-α proteins. The authors also suggested that the peroxisomal product necessary for this antiinflammatory effect in LPS-stimulated macrophages is DHA, leaving to speculation whether peroxisomes produce biolipids to initiate the resolution of inflammation. Of note, DHA has antiinflammatory properties on human primary monocytes and T-helper lymphocytes [60]. Interestingly, in this process, the activity of NF-𝜅B is not affected, suggesting that peroxisomes can regulate the immune cell activation with different strategies that are NF-𝜅B dependent or independent.

The immune regulatory properties of peroxisomes have also been associated to their role in the production of ether lipids that are exclusively produced by peroxisomes in mammals. Ether lipids are particularly abundant in white blood cells; in macrophages and neutrophils, they represent up to 46% of the total phospholipids [61]. Lodhi and colleagues reported that peroxisome-derived phosphatidylcholine and ether lipids are required for neutrophil survival in mice [62]. In another study, Facciotti and colleagues described the requirement of ether lipids for the education, differentiation, and maturation of invariant natural killer (iNKT) cells in the thymus, extending the importance of peroxisomes not only to the innate, but also to the adaptive immune cell differentiation processes. The development and maturation iNKT relies on the recognition of lipid self-antigens presented by the cell-surface molecule CD1d in the thymus [63]. The authors found that mice deficient in the peroxisomal enzyme glycerophosphate O-acyltransferase (GNPAT), essential for the synthesis of ether lipids, showed a significant alteration in the thymic maturation of iNKT cells and fewer iNKT cells in both the thymus and peripheral organs, which confirmed the role of ether-bonded lipids as iNKT cell antigens. Thus, peroxisome-derived lipids are nonredundant self-antigens required for the generation of a full iNKT cell repertoire [63] and essential for cells of the adaptive immune system.

## Peroxisome–pathogen interactions

Peroxisomes can also be targeted by some bacteria and viruses to escape immune responses. West Nile and dengue virus (flaviviruses) infections were shown to trigger peroxisomal biogenesis inhibition [64]. The peroxisome loss is caused by the capsid protein-dependent sequestration and degradation of the peroxisomal biogenesis factor PEX19, which explains why the induction of type III interferon is impaired in cells infected by these viruses. The N-terminal protease of pestivirus localizes to peroxisomes, and this localization inactivates the transcription factor IRF3, one of the main regulators of interferon production [65]. Likewise, the interaction between the human immunodeficiency virus protein, negative regulatory factor, and the peroxisomal enzyme Acyl-CoA Thioesterase 8 (ACOT8), led to a down-regulation of the major histocompatibility complex I, limiting T-cell activation necessary to eliminate infected cells [66–68].

Another study reported by Boncompain and colleagues demonstrated that the bacterium *Chlamydia trachomatis*, an obligate intracellular pathogen responsible for millions of cases of sexually transmitted infections, relies on peroxisomes of the cells to support its metabolism. The study demonstrated that peroxisomes are imported into the *Chlamydia*-contained phagosome in infected cells, and, although the organelle is dispensable for bacterial replication, it seems to be essential for the production of exclusive metabolites, such as plasmalogens, for this bacterium [69]. These emerging cases of pathogens exploiting peroxisomes open new avenues of investigation of peroxisomes as potential therapeutic targets to manipulate host–pathogen interactions for the survival of the host.

## Conclusions

**Fig 1:**
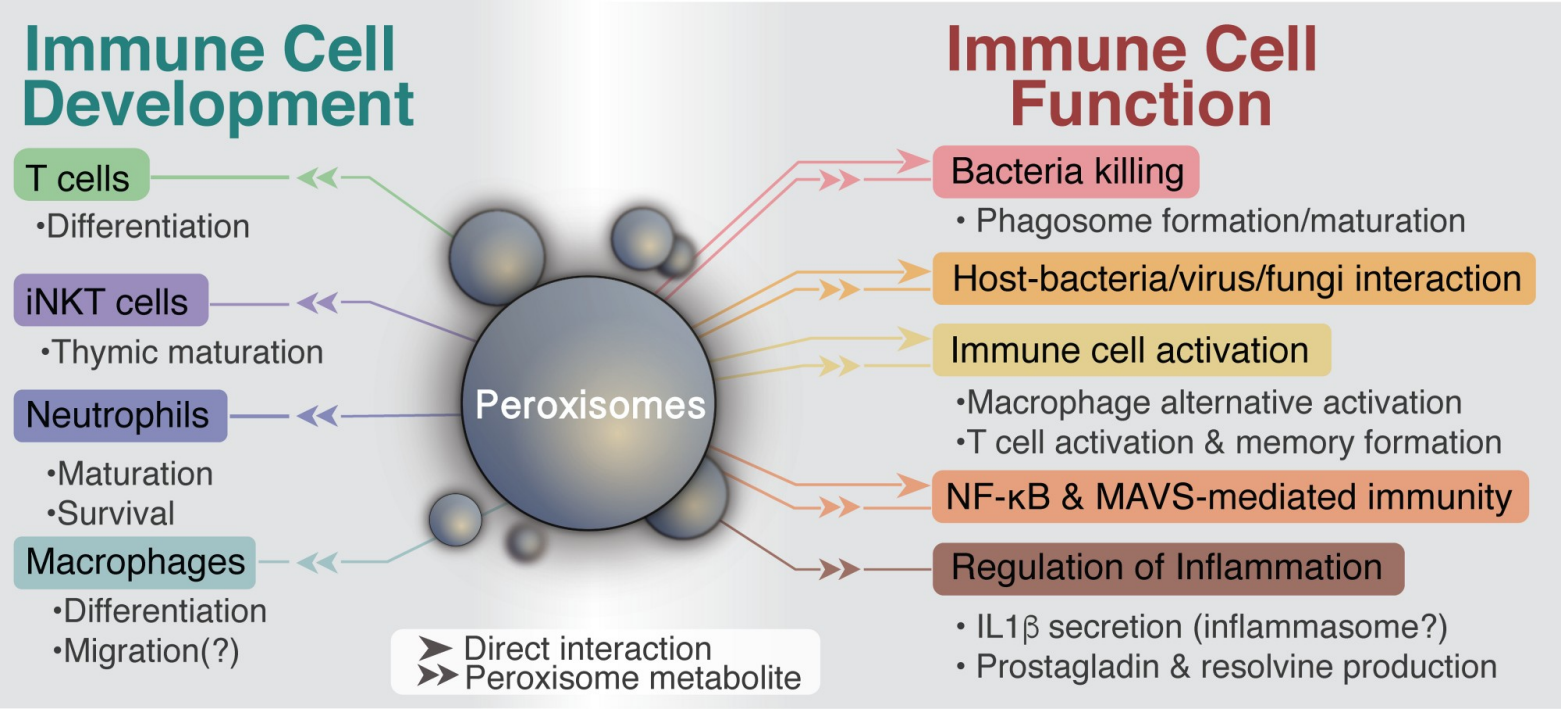
Diagram summarizing the requirements for peroxisomes in modulating host–pathogen interactions.

Immune disorders encompass a wide spectrum of human diseases with an ever-increasing impact on health. In 1970, the World Health Organization classified for the first time “primary immune deficiencies” as a small group of diseases characterized by recurrent or chronic infections, autoimmunity, allergy, inflammation, or cancer as a consequence of genetic alterations affecting the immune system [70]. To date, over 400 characterized immune deficiencies present a worldwide incidence of 1 in 10,000 [71]. There is therefore a pressing need to further characterize the underlying networks that govern immune cell functions in both health and disease to understand and correct immune disorders. The importance of peroxisomes in immunity and inflammation has become clear in the past 10 years (**Fig 1**). The role of peroxisomes in modulating cellular and extracellular fatty acids appears to explain their role as metabolic regulators of various immune functions. However, several peripheral metabolites and pathways are also required to shape the complexity of immune cell development, activation, and inhibition [4], and some of these rely, at least in part, on peroxisomal metabolism. For instance, polyamines, which are catabolized by peroxisomes [14], have been reported to be important for T-cell clonal expansion, macrophage alternative activation, and dendritic cell modulation [4].

Future studies to define how peroxisomes regulate discrete immune processes in innate and adaptive immune cells will be an important step towards understanding how cellular systems as a whole operate to generate and control effective immune responses. Also, these types of investigations will be critical to define how peroxisomal immunometabolic activities contribute to the development of immune disorders, the onset of metabolic diseases and chronic inflammation and elucidate the specific role peroxisomes play in host–pathogen interactions. We speculate that future investigations of peroxisomes in immunity will unveil alternative therapeutic targets to treat infections, inborn errors of immunity, and chronic inflammatory diseases.
